# Supplementary material for: Artificial selection improves pollutant degradation by bacterial communities
Source: Nat Commun. 2024 Sep 7;15:7836. doi: 10.1038/s41467-024-52190-z (PMC11380672; doi:10.1038/s41467-024-52190-z)
Supplement: Supplementary file 1 — Supplementary Information [file 41467_2024_52190_MOESM1_ESM.pdf]

Supplementary figures and tables

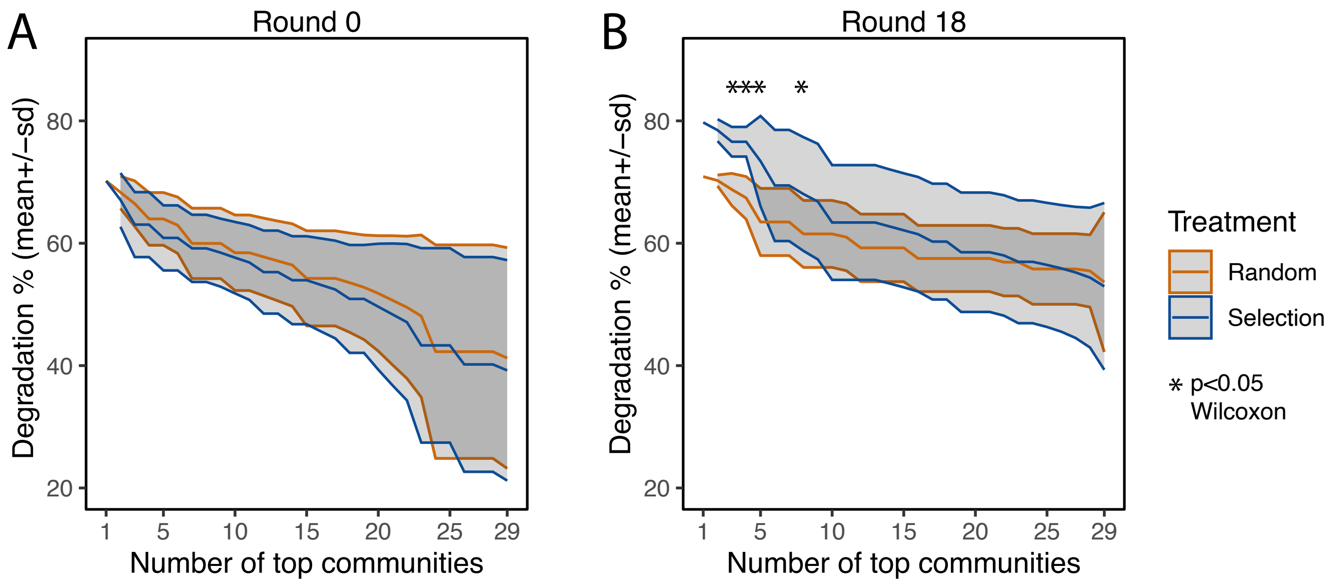

Fig. S1. Justification for why we chose to compare the top 5 communities between treatments. Both plots show the mean±std (central and shaded areas) of the degradation percent of the top x communities in round 0 (A) and round 18 (B). Naturally, the fewer communities we take, the smaller the standard deviation becomes. While in round 0 there is no difference between the treatments, at round 18, the selection treatment becomes significantly better (Wilcoxon rank sum exact test, n=x, $p < 0.05$) only when we take the top few communities, indicated with asterisks. The noise is uninteresting from the perspective of an optimization algorithm, as what we are striving toward is to find the very best community. However, this analysis does suggest that we should work to reduce the noise, for example by lowering the fraction of communities receiving migrants.

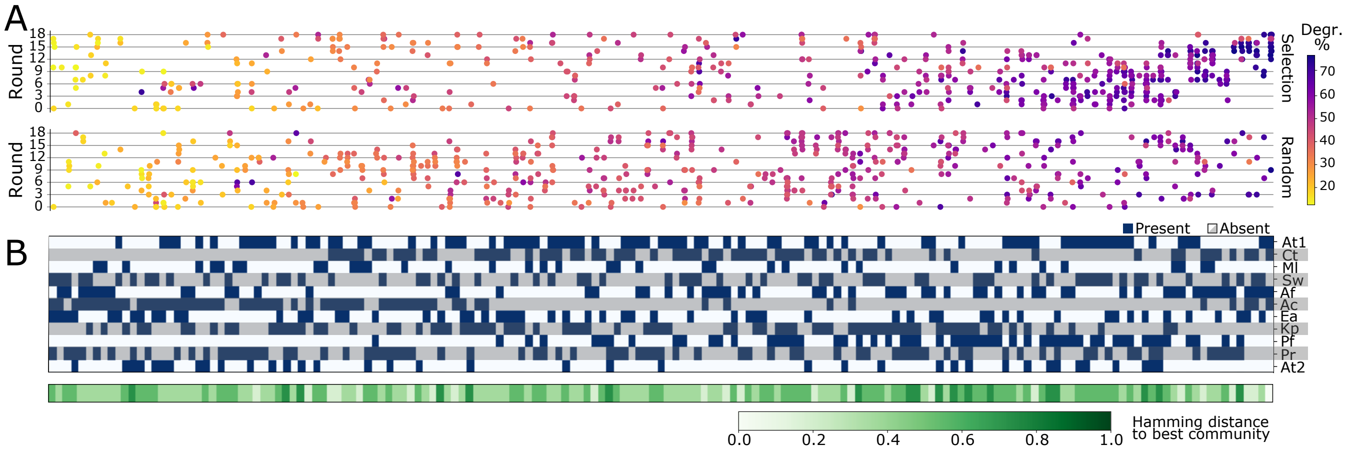

Fig. S2. A) Community composition (x-axis) vs. degradation score (hue, color bar) for all 167 tested communities over the 18 rounds of selection (y-axis) in the selection (top panel) and random (bottom panel) treatments. The x-axis is ordered by increasing degradation scores (averaged over all instances of the same species composition). Note that these are degradation percentages, not final community scores (extinctions not considered). B) Community composition corresponding to panel A, showing the presence (dark blue) or absence (white/grey) of each species, and illustrating the difference in composition by the Hamming distance (i.e. the number of substitutions needed to transform a given community to another) to the community with the highest degradation score at the bottom. This figure overlaps with Fig. 1C, D in the main text, but includes all 167 tested communities as opposed to only the best third (56) of all communities tested.

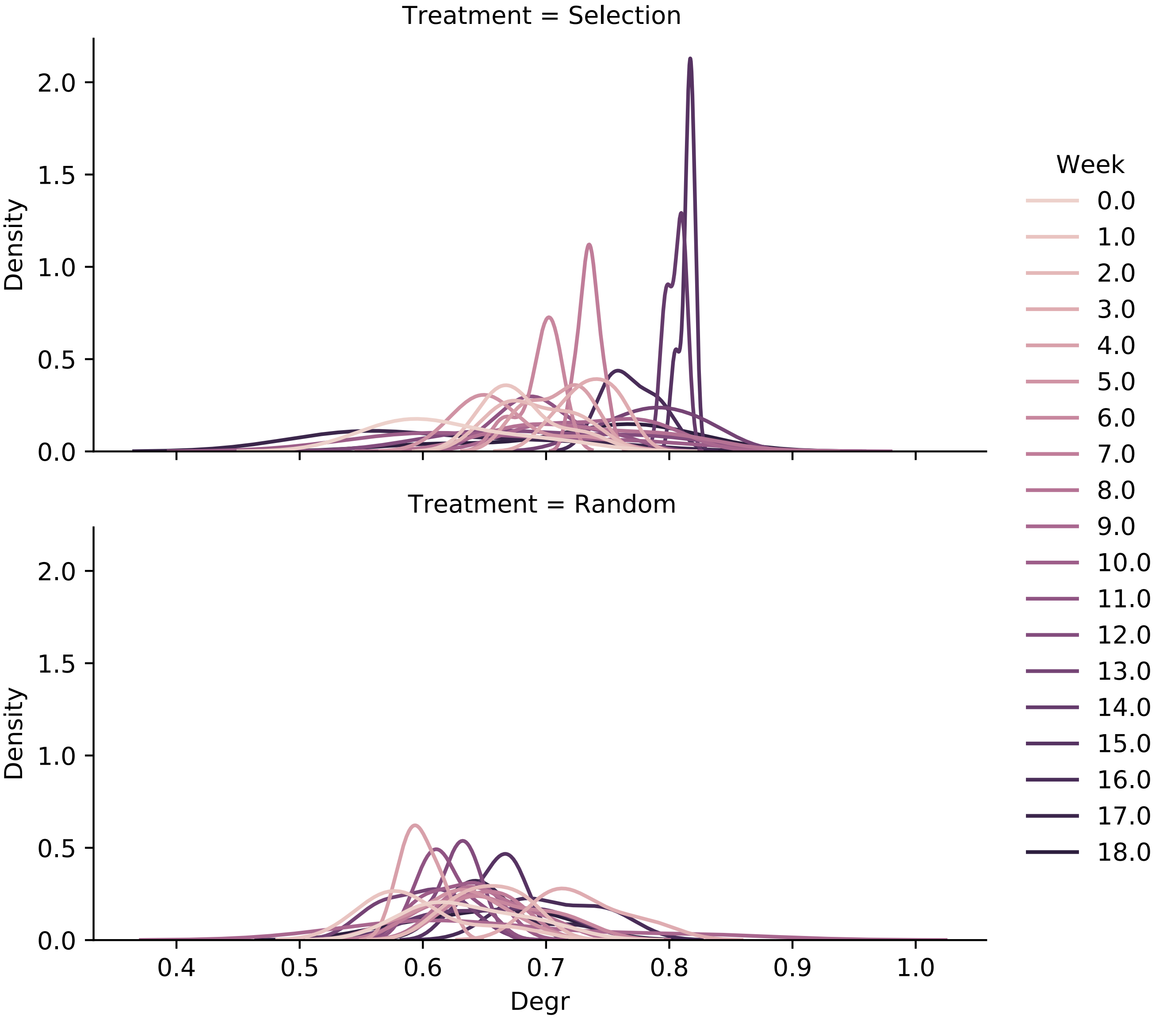

Fig. S3. Distribution (Gaussian kernel density estimates) of degradation scores (x-axis, “Degr”) of all 30 communities, colored by week. Top row: selection treatment, bottom row: random control.

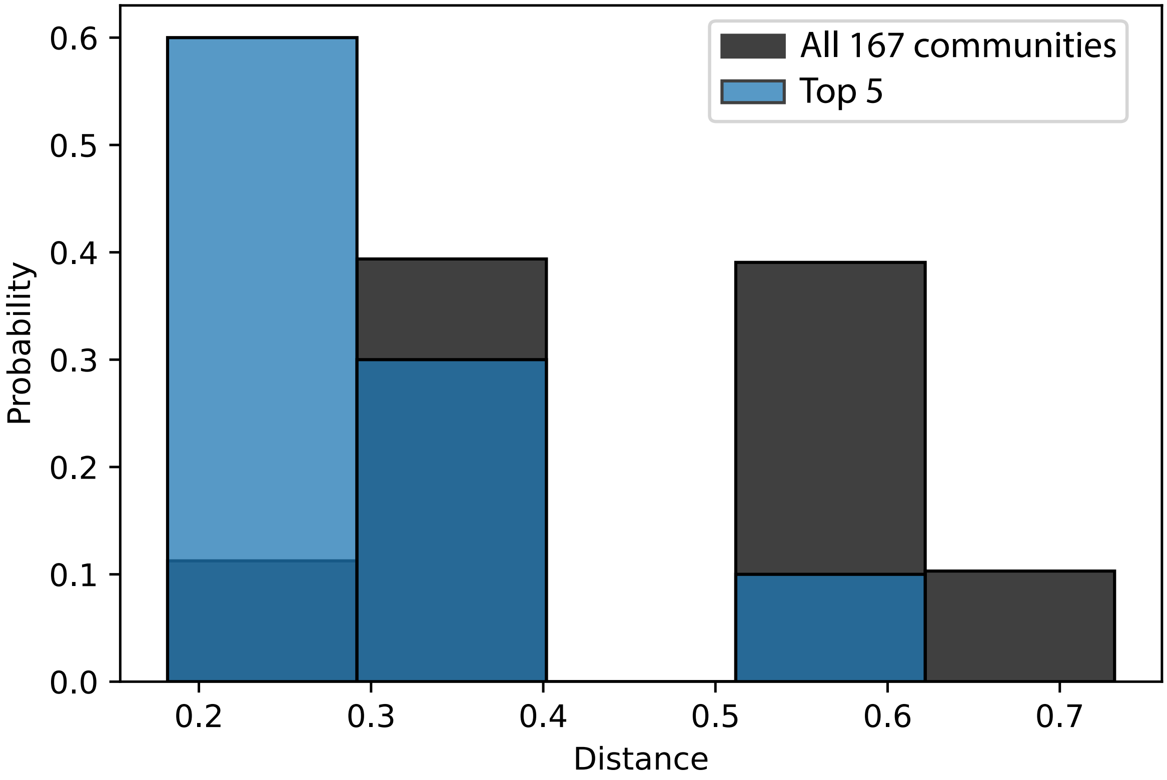

Fig. S4. Distribution of Hamming distance between all pairs of 167 communities in our experiment (black) and between the pairs of the 5 communities with the highest average degradation scores (blue). The distributions are transparent and placed on top of each other.

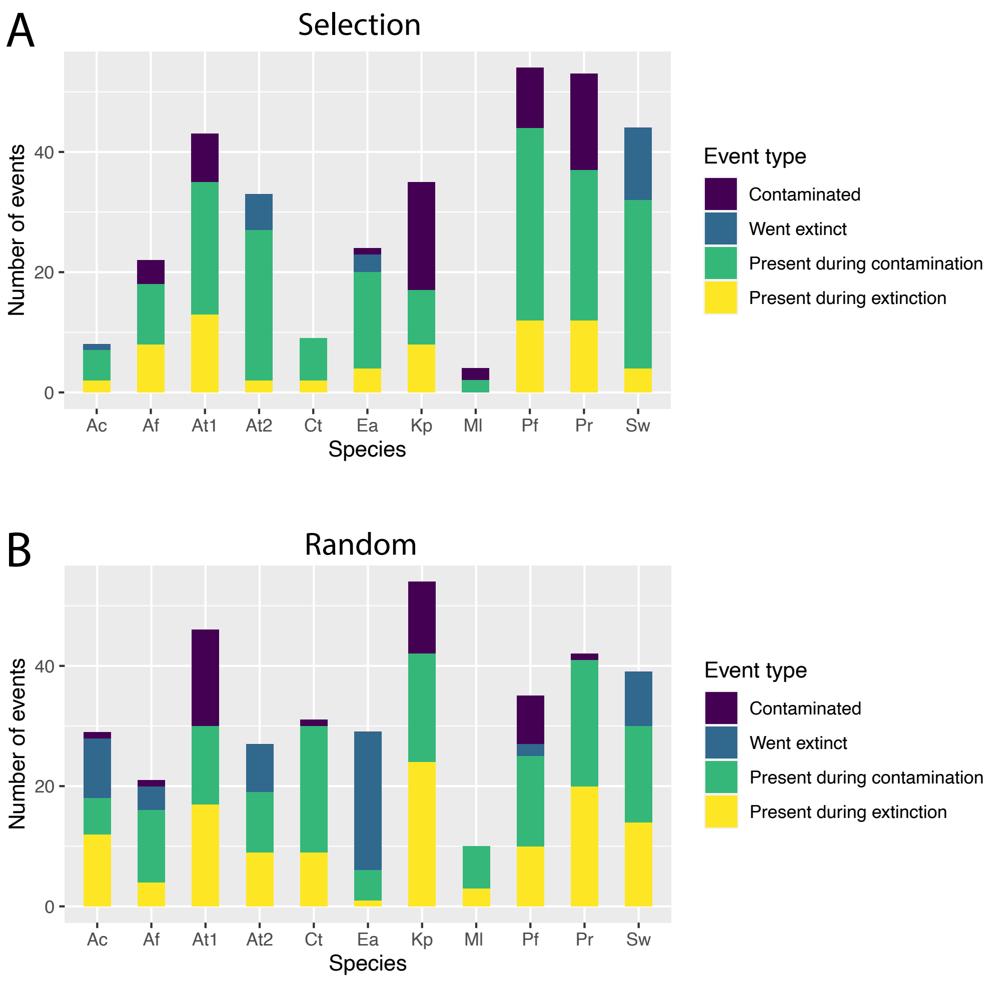

Fig. S5. Species' involvement in extinction and contamination events during the artificial selection experiment in A) the selection or B) the random treatment. We count the number of times a given species went extinct, contaminated a community, was present when an extinction occurred, or was present when a contamination occurred. Data over rounds shown in Tables S1. Species abbreviations are as listed in Table 1.

Round	Community composition	Extinct species
0	Sw_Kp_Pf_Pr	Sw
1	At1_Pf_Pr_At2	At2
1	At1_Pf_Pr_At2	At2
1	Sw_At1_Pf_At2	At2
2	At1_Pf_Pr_At2	At2
2	Sw_At1_Kp_Pf	Sw
2	Sw_At1_Ea_Pr	Ea
3	Sw_Kp_Pf_At2	Sw
3	Sw_Af_Kp_Pf	Sw
3	Sw_At1_Pr_At2	At2
4	Sw_At1_Pr_At2	Sw_At2
7	Sw_At1_Ea_Pf	Sw
8	Sw_At1_Af_Ea	Sw
9	Sw_At1_Ea_Pr	Sw_Ea
9	Sw_At1_Af_Ea	Sw
13	Ea_Kp_Pf_At2	Ea
15	Sw_Af_Ac_Kp*	Ac
16	Sw_Ct_Af_Ac	Sw
16	Sw_Ct_Af_Ac	Sw
18	Sw_At1_Kp_Pf	Sw

Round	Community composition	Extinct species
0	Sw_At1_Ea_Pr	Sw
0	At1_Ml_Pr_At2	At2
1	Sw_Ac_Ea_Kp*	Ea
1	At1_Ml_Pr_At2	At2
1	Sw_At1_Ac_Ea	Sw_Ea
1	Sw_Ac_Ea_At2*	Ea
1	At1_Ac_Ea_At2	Ea
2	Sw_Ac_Kp_At2*	Ac
2	At1_Ac_Ea_At2	Ea
4	Ct_Ea_Kp_Pf	Ea
4	Sw_Ac_Pr_At2*	At2
5	Sw_Ac_Pr_At2*	Ac_At2
5	Sw_Ac_Kp_Pr*	Ac
5	Ac_Ea_Kp_At2*	Ac_Ea
6	Sw_Ct_Kp_Pf	Pf
6	Sw_At1_Ea_Kp	Sw_Ea
6	At1_Ct_Kp_Pf	Pf
6	Sw_Af_Ac_Kp*	Ac
7	Sw_Ea_Pr_At2*	Ea_At2
8	Sw_Ct_Ac_Kp	Sw
8	Sw_Ct_Af_Kp	Sw
8	Sw_Ea_Pr_At2*	Ea_At2
9	Sw_At1_Ac_Kp	Sw
9	Ac_Ea_Pr_At2*	Ea_At2
10	Ac_Ea_Pr_At2*	Ac_Ea
10	Ea_Pf_Pr_At2	Ea
10	Af_Kp_Pf_Pr	Af
11	Ml_Ac_Kp_Pr*	Ac
11	Ea_Kp_Pf_Pr	Ea
11	Af_Ea_Kp_Pf	Ea
11	Ac_Ea_Pr_At2*	Ac_Ea_At2
13	At1_Ac_Ea_Pr	Ea
13	Sw_Ct_Ac_Kp	Sw
14	Sw_Ct_Ea_Kp	Ea
14	Af_Ea_Kp_Pf	Ea
15	At1_Ct_Ea_Kp	Ea
15	Ea_Kp_Pf_Pr	Ea
15	Sw_Af_Kp_Pr*	Sw_Af
16	Sw_Af_Ac_Pr*	Af_Ac
16	Sw_At1_Pf_At2	Sw
16	Ct_Ea_Kp_Pf	Ea
17	Sw_Af_Ac_Pr*	Af_Ac
18	Sw_Ac_Ea_At2*	Ea

Table S1. Extinctions occurring in the selection (left) and random treatment (right). Asterisks mark communities that did not have any strong-growing species (At1, Ct, Pf), which tend to positively affect weaker growers (Fig. 4C). These communities may experience species extinction because they are missing these species, rather than through competition. Species abbreviations are as listed in Table 1.

Treatment	Type of interaction change	number
Random	Not significant in ancestor, now negative	3
Random	Negative in ancestor, now not significant	2
Random	Positive in ancestor, now not significant	2
Selected	Not significant in ancestor, now negative	1
Selected	Negative in ancestor, now not significant	3
Selected	Positive in ancestor, now not significant	2

Table S2. Counts of interaction changes from Fig. 4 where we compare the ancestral to the evolved interactions. “Now” refers to the interactions between the isolates evolved under the respective treatment for 18 rounds. A total of 46 interactions were measured and could have changed in each treatment. Instead, we observe only 7 changes in the random treatment and 6 changes in the selection treatment.

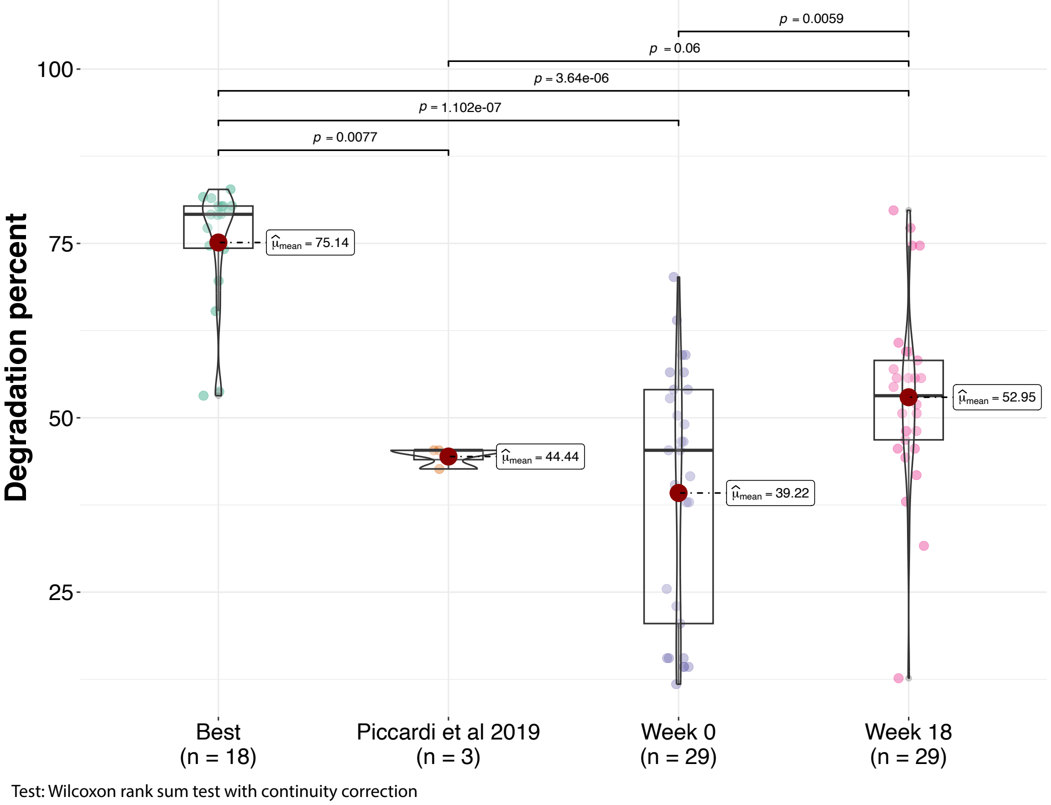

Fig. S6. Comparing the performance of the best community we found (“Best”) to the community studied in our previous work (32). We also include the 29 communities in round 0 of the selection treatment and round 18. Statistical comparison used a Wilcoxon rank sum test.

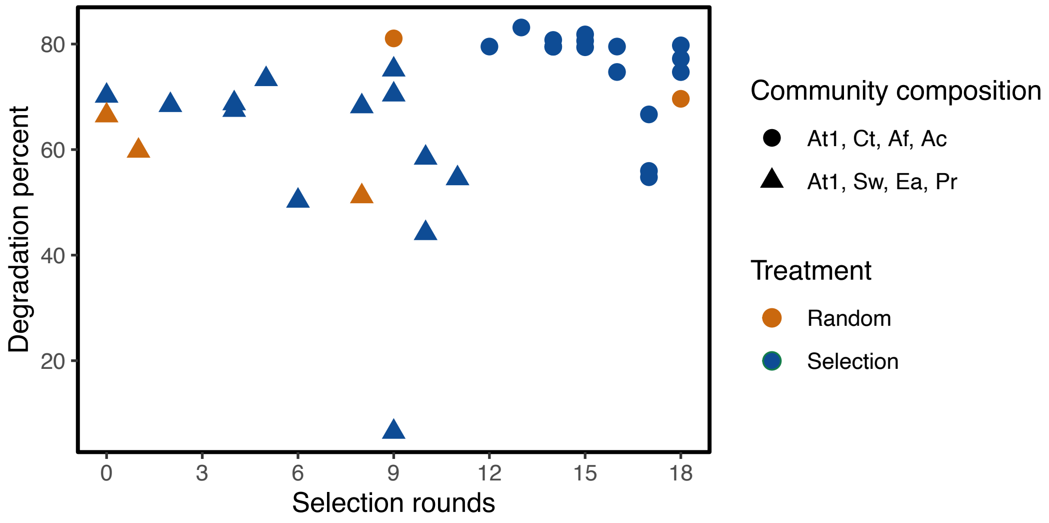

Fig. S7. Simulating how propagule selection would have performed: we plotted the degradation percent of the best-performing community at round 0 over all the rounds in which it appeared in both the selection and random treatments. We also plot all instances of the winning community with our approach. Neither of these communities improve in degradation score over time, but our winning community performs significantly better than the best community from round 0 (59.57 $\pm$ 16.83% versus 75.45 $\pm$ 8.43%, Wilcoxon p=0.0002). Species abbreviations are as listed in Table 1.

Species	Media name	Base medium	Base [g] for final vol. 300 ml	Antibiotic	Antibiotic conc. [µg/ml]
At1	LB+KAN100 +SUL14.25+TRI0.75	LB	12	KAN	100
				SUL	14.25
				TRI	0.75
Sw	MSA+NA15	MSA	33.3	NA	15
Ct, At2	Bru+SS2000+RIF50	Brucella	12.3	SS	2000
				RIF	50
Ml	NutrAgar+FO75 +AmB5+LEX8 +CST5+NA30	Nutrient Agar	8.4	FO	75
				AmB	5
				LEX	8
				CST	5
				NA	30
Af	LB+STR200 +THY100	LB	12	STR	200
				THY	100
Ac	MMChromo+NA60	MMChromo	14.74	NA	60
Ea	MRS+KAN25	MRS	18.6	KAN	25
Kp	KIA+CAR100	KIA	12.24	CAR	100
Pf	Cet+CAR50-Gly	Cetrimide	14.01	CAR	50
			no glycerol		
Pr	PIA+TET15	PIA	13.5	TET	15
			6 ml glycerol		

Table S3. Recipes for selective media. All media were prepared to a total volume of 300ml. Abbreviations are as in Table S4.

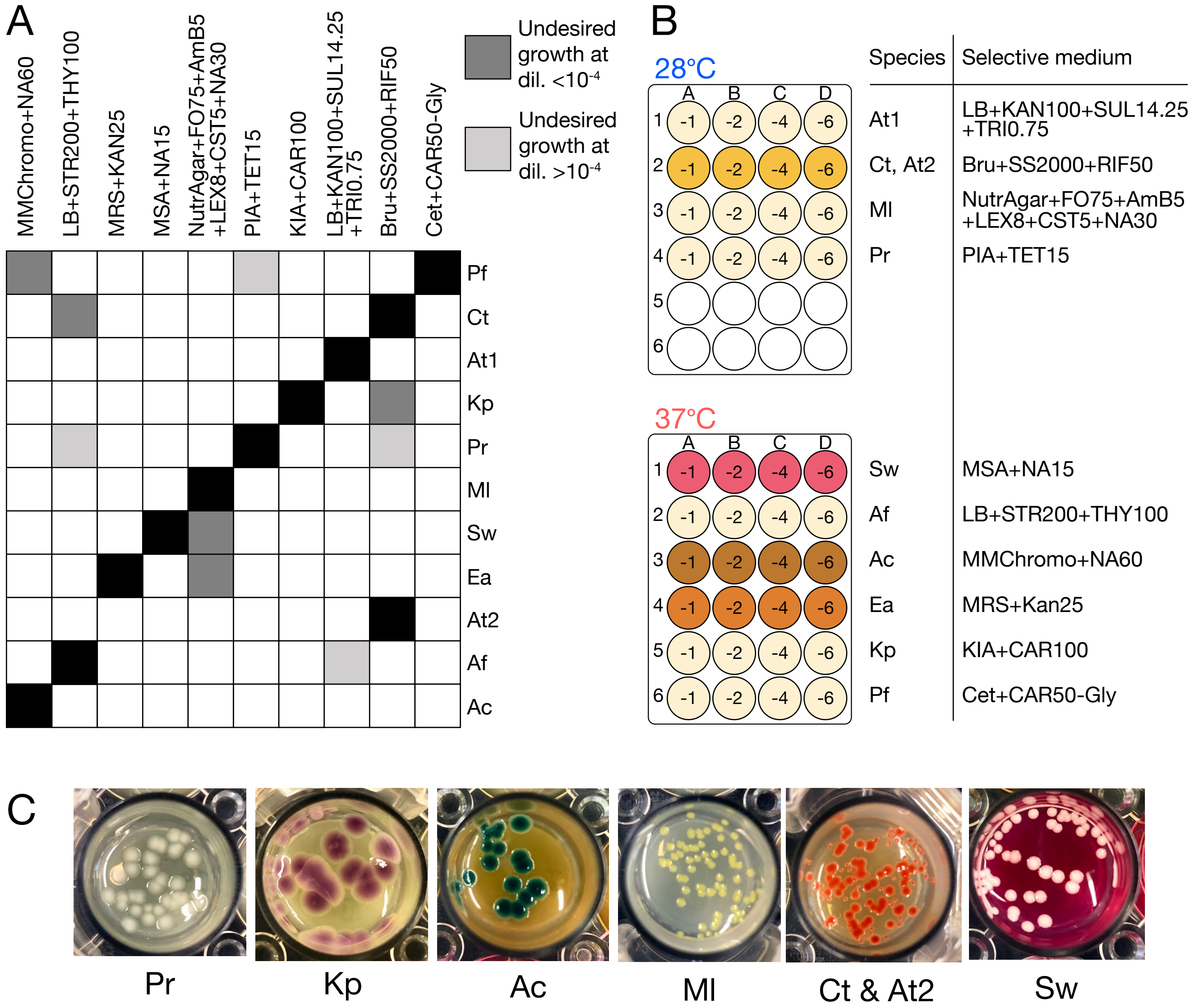

Fig. S8. Selective media for 11 species. (A) Black squares indicate no inhibition of a given species on the corresponding medium, dark grey squares indicate substantial growth (visible at dilution 10^{-4} or at higher dilutions), light grey indicates minor growth (visible at dilution 10^{-4} or at lower dilutions). We avoid combining species with dark grey squares but ignore the light grey ones and just avoid picking colonies from a low dilution. Nevertheless, this can explain some contamination patterns (Fig. S5). Species abbreviations are as listed in Table 1. Other abbreviations as in Table S4. Recipes for selective media are shown in Table S3. (B) 2×20 24-well plates were prepared in advance to contain 1.5ml per well of each selective medium in quadruplicate. We selected 20 communities to plate (10 from selection and 10 from random treatment), diluted each in 96-well plates containing PBS and inoculated 25 μl droplets of the corresponding dilutions into the wells as indicated above (dilutions -1, -2, -4 and -6). This gave us an approximate idea of the population sizes of each species in each of the 20 plated communities. (C) Six selective media with the seven species that could easily be distinguished on them growing in 24-well plates. As Ct and At2 could not be distinguished, we never combined them in the same community.

Abbreviation	Full name
KIA	Klebsiella Isolation Agar (Sigma 90925)
MMChromo	Miller and Mallinson ChromoSelect Agar (Sigma 00563)
PIA	Pseudomonas Isolation Agar (BD 292710)
LB	Luria Bertani Agar (BD 244520)
Bru	Brucella Agar (Sigma 18795)
Cet	Cetrimide Agar (Sigma 22470)
NutrAgar	Nutrient Agar (Oxoid CM0003)
MRS	De Man, Rogosa, Sharpe Agar (Oxoid CM0361)
MSA	Mannitol Salt Agar (Oxoid CM0085)
RIF	Rifampicin
KAN	Kanamycin
SUL	Sulfamethoxazole
TRI	Trimethoprim
AmB	Amphotericin B
LEX	Cephalexin
CST	Colistin
NA	Nalidixic Acid
TET	Tetracyclin
FO	Fosfomycin
STR	Streptomycin
THY	Thymidine
CAR	Carbenicillin
SS	Sodium Selenite

Table S4. Abbreviations used in medium ingredients and chemical sources.
