# Supplementary material for: Artificial selection improves pollutant degradation by bacterial communities
Source: Nat Commun. 2024 Sep 7;15:7836. doi: 10.1038/s41467-024-52190-z (PMC11380672; doi:10.1038/s41467-024-52190-z)
Supplement: Supplementary file 3 — Reporting Summary [file 41467_2024_52190_MOESM3_ESM.pdf]

nature portfolio

Corresponding author(s): Mitri, Sara

Last updated by author(s): Aug 14, 2023

Reporting Summary

Nature Portfolio wishes to improve the reproducibility of the work that we publish. This form provides structure for consistency and transparency in reporting. For further information on Nature Portfolio policies, see our Editorial Policies and the Editorial Policy Checklist.

Statistics

For all statistical analyses, confirm that the following items are present in the figure legend, table legend, main text, or Methods section.

n/a	Confirmed	
☐	☒	The exact sample size (*n*) for each experimental group/condition, given as a discrete number and unit of measurement
☐	☒	A statement on whether measurements were taken from distinct samples or whether the same sample was measured repeatedly
☐	☒	The statistical test(s) used AND whether they are one- or two-sided<br>*Only common tests should be described solely by name; describe more complex techniques in the Methods section.*
☐	☒	A description of all covariates tested
☐	☒	A description of any assumptions or corrections, such as tests of normality and adjustment for multiple comparisons
☐	☒	A full description of the statistical parameters including central tendency (e.g. means) or other basic estimates (e.g. regression coefficient) AND variation (e.g. standard deviation) or associated estimates of uncertainty (e.g. confidence intervals)
☐	☒	For null hypothesis testing, the test statistic (e.g. *F*, *t*, *r*) with confidence intervals, effect sizes, degrees of freedom and *P* value noted<br>*Give P values as exact values whenever suitable.*
☒	☐	For Bayesian analysis, information on the choice of priors and Markov chain Monte Carlo settings
☒	☐	For hierarchical and complex designs, identification of the appropriate level for tests and full reporting of outcomes
☐	☒	Estimates of effect sizes (e.g. Cohen's *d*, Pearson's *r*), indicating how they were calculated

Our web collection on statistics for biologists contains articles on many of the points above.

Software and code

Policy information about availability of computer code

Data collection	All code associated with this manuscript can be found on Zenodo/Github at DOI: 10.5281/zenodo.1278580.
Data analysis	We have provided details on the software versions (R and Python) used to analyse the data.

For manuscripts utilizing custom algorithms or software that are central to the research but not yet described in published literature, software must be made available to editors and reviewers. We strongly encourage code deposition in a community repository (e.g. GitHub). See the Nature Portfolio guidelines for submitting code & software for further information.

Data

Policy information about availability of data

All manuscripts must include a data availability statement. This statement should provide the following information, where applicable:

- Accession codes, unique identifiers, or web links for publicly available datasets
- A description of any restrictions on data availability
- For clinical datasets or third party data, please ensure that the statement adheres to our policy

The raw data generated in this study have been deposited in the Zenodo database under DOI: 10.5281/zenodo.12784769.

Research involving human participants, their data, or biological material

Policy information about studies with human participants or human data. See also policy information about sex, gender (identity/presentation), and sexual orientation and race, ethnicity and racism.

Reporting on sex and gender	Not applicable
Reporting on race, ethnicity, or other socially relevant groupings	Not applicable
Population characteristics	Not applicable
Recruitment	Not applicable
Ethics oversight	Not applicable

Note that full information on the approval of the study protocol must also be provided in the manuscript.

Field-specific reporting

Please select the one below that is the best fit for your research. If you are not sure, read the appropriate sections before making your selection.

☐ Life sciences ☐ Behavioural & social sciences ☒ Ecological, evolutionary & environmental sciences

For a reference copy of the document with all sections, see nature.com/documents/nr-reporting-summary-flat.pdf

Ecological, evolutionary & environmental sciences study design

All studies must disclose on these points even when the disclosure is negative.

Study description	This study tests a selection algorithm and applies it to finding a microbial community able to efficiently degrade industrial pollutants. It consists of two treatments: a selection and a control treatment, each consisting of 29 microcosms. We conduct 18 rounds of selection, with 29 microcosms for each treatment at each round (selection experiment). For each community we measure its degradation score and the population sizes of its different members. We then analyse which species were more likely to be present in winning communities than by chance, and within-community diversity. We then conducted follow-up experiments to understand different properties of the species and how they interact with one another (which species significantly enhance or inhibit the growth or degradation of another). These follow-up experiments also evaluated whether these properties and interactions changed over time and between treatments.
Research sample	In the selection experiment, we chose to use 29 tubes per treatment because that allowed us to generate sufficient combinations of 4 species from a pool of 11. The two treatments had identical sample sizes. Ideally we would have used 30 tubes per treatment (to have a round number) but we didn't because the racks on which we placed our tubes had 30 slots and we needed abiotic controls. To compare the behaviors of different species and different strains (evolved or ancestral) and measure their interactions in follow-up experiments, we performed growth experiments in three or four replicates, which is standard in microbiology, as effect sizes are often quite large.
Sampling strategy	See above.
Data collection	Data were collected by different people in different experiments. AH performed the selection experiment and collected data weekly on degradation rates and population sizes. Follow-up experiments were conducted by 2 co-authors, GA and BV who measured degradation rates and population sizes in the presence or absence of other species (to measure interactions). We took the experimenter into account as a random variable in statistical tests and only used data from the same experiment when estimating interactions.
Timing and spatial scale	The selection experiment was conducted over 18 consecutive weeks. Follow-up experiments were conducted by GA during 8 consecutive weeks. We then realized that one of our selective media was not allowing us to count population sizes as expected. BV repeated the experiments over 4 consecutive weeks approximately 18 months later.
Data exclusions	We excluded data collected by GA where we noticed that the selective plates were not working. Selective media are used to count population sizes of one species in the absence of another (they kill one species, but should not affect the species of interest). Unfortunately, after the experiments were finished, we realized that the selective plates were also killing the species of interest. We could tell because we had planned a non-selective medium (where all species grow) on the side and could detect a repeatable difference. We excluded these data and repeated the experiment with more replicates on non-selective medium, which gives lower precision but nevertheless allowed us to estimate population sizes.
Reproducibility	The selection experiment inherently tests the same species combinations many times, particularly if they perform well. This allowed us to gain confidence in the measurements we were making and to detect sources of variability (e.g. removing one species reduced this variability).
Randomization	The positions of communities within a rack were random, these positions were generated using a script that the experimenter ran

each week that indicated where to put each tube. Further randomization procedures performed by the script are described in the methods section (e.g. when species are replaced in a community, they are chosen at random but favoring species that are not present in the meta-community).

Blinding

AH who was conducting the selection experiment was initially not told which treatment was selection and which was the random control. However, we quickly realized that this was futile, as it was obvious that the script chose the best communities for her to plate in one treatment but not the other. Blinding in this part was therefore not possible. GA who conducted the follow-up experiments was not aware of which species and species pairs should degrade well or not (she was not involved in the selection experiment).

Did the study involve field work? ☐ Yes ☒ No

Reporting for specific materials, systems and methods

We require information from authors about some types of materials, experimental systems and methods used in many studies. Here, indicate whether each material, system or method listed is relevant to your study. If you are not sure if a list item applies to your research, read the appropriate section before selecting a response.

Materials & experimental systems

n/a	Involved in the study	
☒	☐	Antibodies
☒	☐	Eukaryotic cell lines
☒	☐	Palaeontology and archaeology
☒	☐	Animals and other organisms
☒	☐	Clinical data
☒	☐	Dual use research of concern
☒	☐	Plants

Methods

n/a	Involved in the study	
☒	☐	ChIP-seq
☒	☐	Flow cytometry
☒	☐	MRI-based neuroimaging
